# Parental Stress Assessment with the Parenting Stress Index (PSI): A Systematic Review of Its Psychometric Properties

**DOI:** 10.3390/children9111649

**Published:** 2022-10-28

**Authors:** Mercedes Ríos, Sara Zekri, Yurena Alonso-Esteban, Esperanza Navarro-Pardo

**Affiliations:** 1 Centro de Estudios Marni (Servicio de Orientación), 46019 Valencia, Spain; 2 Department of Developmental and Educational Psychology, Universitat de Valencia, 46010 Valencia, Spain; 3 Department of Psychology and Sociology, University of Zaragoza Campus Teruel, 44003 Teruel, Spain

**Keywords:** parental stress, assessment, parenting stress index (PSI), psychometric properties

## Abstract

Parental stress is a construct related to people’s perception of difficulties and feelings of not being able to cope with the demands of parenting. This construct is often experienced as a negative or aversive response to parental obligations, and the available evidence also suggests that excessive parenting stress reduces the use of positive parenting behaviors and are related to dysfunctional parenting. Different instruments exist to assess parental stress. This article is part of a project to translate and adapt the Parenting Stress Index (PSI), fourth edition, in its two forms (full and short). The aim of this research is to identify the psychometric indicators obtained by this instrument and to review the evidence they can provide. Method: Following the PRISMA guide (Preferred Reporting Items for Systematic Reviews and Meta-Analyses), the studies related to the PSI were identified in different databases (ERIC, PsycArticles, PubMed, Scopus and Web of Science). Results: The screening process resulted in 16 articles; four have analyzed the psychometric properties of the PSI-4 and the rest have studied the PSI-3. Although version 4 was published in 2012, the studies are scarce. However, they follow the line noted in the previous short version, a high internal consistency and a factor structure of three factors. Conclusions: The difficulties of working with a measurement instrument with 101 items means that the full version of the PSI has been little studied, except in translation and linguistic adaptation studies.

## 1. Introduction

Parental stress has been defined as stress related to parenting, as opposed to other forms of stress experienced by parents, such as economic hardship, work or academic stress or negative life events. The study of that construct is of great clinical and research interest because it has been linked to negative parenting characteristics, such as low levels of parental warmth, unhealthy parenting styles, harsh discipline and potential child neglect or abuse [1,2]. The impact of high levels of parental stress can have a direct impact on a child’s socio-affective and cognitive development [3,4,5]. Parenting stress has also been associated with other disorders such as parental anxiety and depression [6,7,8], marital conflict [9,10], poorer physical health [11,12], reduced parenting effectiveness [13,14] and as a source, origin or reinforcement of children’s behavioral problems [15,16,17,18]. From toddlerhood to adolescence, increased parental stress can create a chaotic family environment that would contribute to an increase in children’s behavioral problems [19].

Different instruments exist to measure parental stress with different levels of evidence [20] (e.g., Caregiver Strain Questionnaire (CGSQ; [21]); Family Impact Questionnaire (FIQ; [17]); Parenting Daily Hassles Scale (PDH; [22]); Parental Stressor Scale [23]; Stress Index for Parents of Adolescents (SIPA; [24]); Questionnaire on Resources and Stress (QRS-F; [25]) and Parental Stress Scale (PSS; [26] or related to specific times such as labor or postpartum or preterm birth [27,28,29,30]). The Parental Stress Index (PSI) [31] is one of the most common and widely used instruments for both clinical and research purposes [32,33] especially in its short forms due to its ease of application. The PSI has been used as a gold standard to validate other instruments [24,26]. In specific areas such as neurodevelopmental disorders (e.g., intellectual disability, autism spectrum disorders, hyperactivity, among others), severe or chronic health problems in children, the use of the PSI is very common [34]. There is currently no official version of the PSI (full or short form) available in Spanish, which has motivated the project of translation and adaptation of this instrument of which this article is a part.

The PSI model [31] considers parental stress to be composed of two dimensions: general stress associated with parental demands and stress that is specifically derived from the child’s own demands. This scale assesses areas of dysfunctional parent–child relationships with children between 1 month and 12 years of age, in order to identify dysfunctional relationships [31]. The original PSI [31,35] measures parenting stress perceived by the caregivers. It was a five-point Likert-type scale including 120 items that consisted of two domains: the child and parent characteristics domain (101 items) and the optional stressful life events domain (19 items). Specifically, the 101 items are divided into 47 items for the child domain and 54 items in the parent domain.. The two domains are structured in a total of 13 sub-domains. The PSI-Short Form (PSI-SF) is composed of 36 items [36], which is a direct derivation of the full-length PSI and three-factor structured (Parental Distress, Difficult Child and Parent–Child Dysfunctional Interaction). Since the third edition of the PSI was published in 1995, a considerable body of literature has accumulated evidence that confirmed its clinical utility and good psychometric properties [20]. Given major demographic and family changes in recent decades, a revision was proposed which has resulted in the PSI 4th edition [37]. The changes introduced in the 4th version focus on two fundamental points: (a) in contrast to the lack of specification in the 3rd version, the 4th version evaluates a particular child, and (b) the language has been adapted so that it can be used in the various current family forms (single-parent families, reconstituted families, foster families, homoparental, etc.). Since the first version of the PSI, different studies have been published showing good internal consistency and adequate test–retest reliability, as this instrument has been translated (in their forms and versions) into different languages and validated in different cultural groups (e.g., China [38,39], Portugal [40], France [41], Canada [42], Finland [43] and the Netherlands [44]).

The main goal of the PSI-4 is to address weak items and outdated language without altering the empirically validated and clinically relevant structure in assessing the overall level of parental stress. The main improvements included in the PSI-4 are cultural sensitivity of language in the items, increased internal consistency of the scales and item loading factor in the scales; moreover, norms based on age, mastery level and subscales have been added, and parents have been included in the standardization sample; finally, T-scores have been added to improve interpretation of the PSI [37]. Another significant change in PSI-4 is that the answers are made regarding the stress produced by raising a particular child. Earlier versions and other questionnaires ask about parenting in general. This fact makes this new version more efficient and specific, since raising all children does not generate the same level of anxiety. In this way, PSI-4 allows to identify, at a clinical and non-clinical level, the problem areas and strengths in relation to the child, the parents and the family system in order to be able to carry out the adequate design of a treatment and follow-up plan [37].

### Present Study

Overall, the PSI has become one of the most widely used instruments to measure parental stress across a wide range of families and children (including those with disabilities). Although the PSI has been applied in a variety of studies, few studies have examined its psychometric integrity [20]. So, this article is a systematic review of the literature related to the psychometric properties of the PSI instrument (both third and fourth versions) in its two modalities: long and short form. Our main objective is to identify studies that analyze PSI from a psychometric point of view and cross-cultural studies to review the evidence that these articles can provide in order to prepare the design of the PSI-4 Spanish version.

## 2. Materials and Methods

### 2.1. Search Strategy

**Figure 1:**
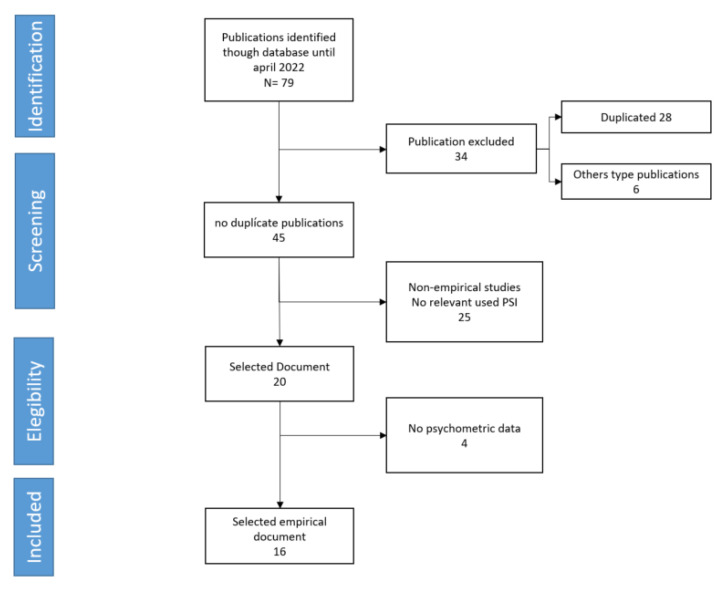
PRISMA flow diagram displaying article selection process.

Following the PRISMA guide (Preferred Reporting Items for Systematic Reviews and Meta-Analyses) (Figure 1), one database has been selected for each adjoining area of knowledge: one for health (PubMed), one for psychology (PsycArticles) and one for education (ERIC) and two general databases (Scopus and Web of Science) All searches included the English key terms parenting, stress and index. The search was performed by accessing all the databases using the online search interface TROBES of the Documentation and Library Service of the University of Valencia (Spain).

### 2.2. Inclusion and Exclusion Criteria

The search was restricted to studies published between 2012 and 2022, so all studies were developed after the publication of the PSI-4 [37]. We excluded all publications that were not published in publishers following blind peer review (e.g., books, book chapters, working papers, conference proceedings and master’s theses). Review studies or clinical trials were also excluded if they did not provide psychometric data on the PSI-4.

### 2.3. Procedure

A total of 79 articles were found in the initial keyword search. After removing duplicate articles, 45 articles remained. Subsequently, the articles were screened by reading the titles, selecting only those in which all the keywords (parenting, stress and index) appear in the title. Finally, 25 articles were excluded in the screening phase. The remaining articles were examined independently by two researchers, who agreed to exclude four more because they did not provide psychometric data. The remaining 16 articles were used to prepare the present paper. Initially, the aim was to include articles that examined the psychometric properties of the latest version (PSI-4); however, given the small number of identified studies of the 4th version, and understanding that it was relevant to compare the psychometric data with those of the 3rd version, it was decided to include the items of the 3rd version as well. All articles were included regardless of the form (SF or LF) studied. The complete reading and selection of the articles was carried out by the first two authors of this work and supervised by the last two of the authors.

## 3. Results

Table 1 presents a brief description of the main characteristics of each of the 16 selected articles, including the author(s), type of study and sample and different psychometrics data (e.g., internal consistency; test–retest reliability, convergent validity and construct validity).

Luo et al. [45] explained that, despite its popularity, there is a lack of consensus on the factor structure of the PSI-SF (e.g., [32,46] As noted above, very few studies have studied the PSI-SF in the Spanish population. All of them are from version 3, although they were developed many years after version 4 was published; this is justified by the lack of an official 4th version in Spanish.

Of these, only five studies have been developed on the Spanish population; the study by [47], which analyses the psychometric properties using the Rasch model of the Spanish version of the PSI-SF, with a sample of 542 participants, male and female, parents of children with intellectual disabilities; the research by [48] studying the factor structure and psychometric properties of the Spanish version of PSI-SF with a sample of 309 mothers (203 with difficulties managing their children’s behavior and 106 from the general population); and finally, the study by [49] on the validity of the PSI-SF in a sample of at-risk mothers with a sample of 149 participants. 

Four studies have analyzed psychometric properties of the PSI-4. Two of them have focused on the short version of the instrument [41,57] and the other two on the long version [58,59].

Barroso et al. [57] conducted a three-moment follow-up study of 58 mothers of preterm infants aged 12–15 months with behavioral problems and low-income Hispanic origin. English and Spanish versions of the PSI-4-SF are used. Overall internal consistency (alpha < 0.91) as well as test–retest stability (r < 0.58) and ICC intraclass correlations (<0.77) show adequate psychometric performance. The second study [41] presents the validation of the French version of the PSI-4-SF with a sample of 318 participants (cross-sectional design with three samples collected from the background population and a sample of parents who have a child with a chronic illness). The data show that the internal consistency is adequate (alpha < 0.89); moreover, the CFA developed on the original three factors model allows us to accept this structural hypothesis. 

Given the length of the full version of the PSI-4, it is difficult to replicate the factor structure and, thus, the construct validity. Not surprisingly, no studies of PSI-3 LF were found. Çekiç et al. [58] have translated and adapted the PSI-4 long version into Turkish, with a sample of 386 parents of children with psychological problems. The internal consistency is adequate (although the study does not provide the alpha for the total test) in each domain (Child Domain 0.92 and Parents Domain 0.95), decreasing slightly in each of the thirteen subdomains depending on the number of items that compose them, and stability of the measure (test–retest) is adequate (0.78). To test construct validity, a separate CFA was conducted for each of the two domains. The results confirm that each domain is formed by the corresponding subdomains; however, an AFC was not performed on all 101 items to confirm the structure of the two domains as second-order factors.

Pereira et al. [59] developed a Portuguese version with 53 Brazilian mothers of children born preterm. The internal consistency coefficients (alpha) are adequate (in all domains and subdomains above 0.80). Given the size of the sample, it was decided to perform a principal component analysis (PCA with varimax rotation) on the scores of the 13 subdomains, finding a bifactorial structure compatible with the two domains (parents and children).

## 4. Discussion and Conclusions

Over the past few decades, there has been an increase in both clinical and research interest in measuring and understanding the effects of parental stress [20]. Testing the existence of reliable and valid measurement instruments is one of the fundamental aims of this work. If we have these tools, we can consider evaluating the level of effectiveness of our interventions in family, clinical or preventive settings. Although scarce, there is evidence on the value of the PSI in its different versions [31,35,37,60] for measuring parental stress and, thus, identifying dysfunctional parental relationships. Over the last decades, efforts to understand parental stress have steadily increased. Many studies have attempted to analyze the psychometric properties of the PSI, which assesses parent–child problem areas in children between one month and twelve years of age to identify dysfunctional parent–child relationships [35].

Holly et al. [20] provide a comparative study of eight parental stress measurement instruments (considering the PSI and PSI-SF as two different instruments). The frequency of use in the analysis of the found data shows that the PSI (in its two forms) is not only the most widely used tool, but also the one that provides the best evidence on its norms, validity, reliability and usefulness of its measures.

In adaptations of scales to cultural and linguistic environments other than the original one, according to the International Testing Commission (ITC) [61,62], the confirmation guidelines propose to carry out studies on the reliability, validity and metric equivalence between each of the items that make up the test and the dimension they represent. 

Thus, to avoid methodological bias, given that Likert-type items are polytomous, Pearson’s correlation was not used. This statistic requires continuity of the variable and measurement on an interval scale, but polytomous item categories do not meet either of these qualities, so it is necessary to use a factor estimation method suitable for this type of variable. In this case, two very common methods can be used: the unweighted least squares method or the robust weighted least squares method (e.g., [63]). The latter is better; the drawback is that it is only implemented in Mplus [64], but the first method can produce a good approximation of the factor structure of an instrument when performing an EFA. 

A clearly neglected aspect in psychometric studies with the classic test model is the study of the goodness of fit of the number of categories used to obtain information in the items. That is, the classic test model assumes that categories are set by authors regardless of their ability to obtain the most appropriate information from individuals about the measured attribute. However, there are multiple studies showing that the use of fewer or more categories, whether odd or even, produces clear discrepancies in obtaining sensitive information about the measured psychological trait. Nevertheless, for the past four decades, methodology based on Rasch and IRT modelling has made it possible to study the functioning of item categories in detail [65,66].

Factor studies of the long version of the PSI are scarce and methodologically weak, and are generally associated with translation, adaptation and typing studies in which the sample must be sufficiently large. It is within the framework of these studies that the construct validity of the full PSI should be confirmed and the same for the short version. Moreover, most of the found studies have been conducted on clinical or at-risk samples. Sometimes, the factor structure is not replicated in such samples, so it is advisable to conduct validation studies on general population samples, without known problems. It should be understood that parental stress is a construct that manifests a continuum of measurement, so that higher or lower scores can be used as an indicator of dysfunction. The use of clinical samples or samples drawn from the at-risk population to estimate diagnostic validity is appropriate; however, the factor structure of the scales calculated solely from the scales may lead to the erroneous assumption that the scale structure is invalid when, perhaps, what is revealed is only the structure of the sample used, with a specific pathology. 

Consequently, the importance of continuing research on the PSI and developing, if possible, a coherent model for the two forms (LF and SF) should be emphasized. The clinical use of the PSI has shown that it is a useful tool for the early detection of high levels of stress in families and the analysis of its origin (both in clinical and non-clinical samples), which may allow them to improve their coping strategies and skills through the design and creation of preventive and family intervention programmers, as well as individualized psychosocial and educational resources adapted to their circumstances in order to reduce their stress to an optimal level [37,47,67].

## Figures and Tables

**Table 1:** Summary information about Parenting Stress Index articles.

Form	Citation	Population		Reliability				Validity	
		Sample N	Specific Sample	Internal Consistency Cronbach’s α McDonald’s ω ISR	Test–Retest	Inter-Rater Correlations	ICC	Concurrent Validity	Factorial Structure
SF-3	Aracena et al. (2016) [50]	336 dyads mother–child	Clinical (low-income mother–child dyads from 24 health care centers)	GPS = 0.92 PD = 0.81 P–CDI = 0.89 DC = 0.88	NA	NA	NA	GGHQ r = 0.86	EFA Three-factor compatible with the original version
SF-3	Dardas and Ahmad (2014) [51]	N = 184 parents of children with autistic disorder (114 female)	Clinical (parents of children with autistic disorder)	GPS = 0.91	NA	NA	NA	NA	EFA (principal components and varimax rotation) Three-factor compatible CFA
SF-3	Derguy et al. (2020) [52]	N = 370 (5 samples). Parents of children with autism spectrum disorder (73.2% female and 26.8% male)	Clinical (parents of children with autism spectrum disorder) participants in various parents’ support programs on parental stress	GPS = 0.87 PD = 0.86 P–CDI = 0.82 DC = 0.80	NA	GPS = 0.52 PD = 0.56 P–CDI = 0.33 DC = 0.49	GPS = 0.71 PD = 0.73 P–CDI = 0.52 DC = 0.70	HADS PD and anxiety r = 0.71 Global and Anxiety 0.60 Global and Depression 0.50 WHOQOL-BREF R = −0.50 ALES T = 0.50 L = 0.60 C = −0.23	CFA poor confirmation three-factorial structure EFA unweighted least squares (ULS) extraction method and oblimin rotation
SF-3	Emam et al. (2022) [53]	N = 867 parents had at least one child with a disability	Arabic version and trans-country studies (Oman: 380 parents; Saudi Arabia: 300 parents; Qatar: 187 parents)	GPS = 0.7	NA	NA	NA	NA	CFA Three-factor structure supported
SF-3	Gao and Lee (2021) [54]	N = 448 parent–child dyads	Cross-cultural study (Hong Kong: 258 parent–child dyads; Thailand: 190 parent–-child dyads) 15 items reduced version used	ω = 0.71 to 0.78	NA	NA	NA	KPDS r= 0.37 to 0.42 PSDQ r = −0.25 to −0.49	CFA Three-factor structure supported
SF-3	Jenaro and Gutiérrez Bermejo (2015) [47]	N = 542 participants, parents or guardians of children with intellectual disabilities (374 female and 168 male)	Clinical (parents or guardians of children with intellectual disabilities)	ISR = 0.99 PSR = 0.94	NA	NA	NA	NA	NA
SF-3	Lee et al. (2016) [46]	N = 240 caregivers (204 mothers, 7 fathers, 1 parent, 10 grandparents, 13 others)	Clinical (Black and Latino caregivers of children with behavioral difficulties)	NA	NA	NA	NA	CES-D GPS and CES-D r = 0.61 IOWA GPS and IOWA-IO r = 0.26 GPS and IOWA-OD r = 0.45	CFA Three-factor structure supported
SF-3	Luo et al. (2021) [45]	683 mother–father dyads	Non-clinical (Mainland Chinese parents of nonclinical children to develop a psychometrically abbreviated version of the PSI-SF)	GPS 0.86 to 0.87 PD 0.71 to 0.72 P-CDI 0.78 to 0.82 DC 0.78 to 0.79	NA	GPS = 0.96 PD = 0.90 P-CDI = 0.92 DC = 0.93	NA	PBQ Positive parenting −0.21 to −0.42 Corporal punishment 0.40 to 0.46 Overlook 0.20 to 0.36 CES-D r = 0.28 to 0.35 SDQ Emotion 0.25 to 0.44 Hyperactivity 0.16 to 0.39 Prosocial −0.22 to −0.37	EFA Principal axis factor and promax rotation CFA No three-factor structure supported
SF-3	Park and Chae (2020) [55]	N = 114 mothers of children with cerebral palsy	Clinical (mothers of children with cerebral palsy)	GPS 0.91 PD 0.90 P-CDI 0.78 DC 0.83 PSR = 0.92 ISR = 0.95	NA	NA	NA	NA	NA
SF-3	Pérez-Padilla et al. (2015) [49]	N = 149 mothers (109 at-risk mothers and 40 mothers, sample of community families)	Clinical (sample of at-risk mothers)	GPS 0.89 PD 0.79 Child rearing stress 0.85	NA	NA	NA	PSOC 0.48 PLOC −0.34 GGHQ 0.39	NA
SF-3	Rivas et al. (2021) [48]	N = 309 mothers (203 with difficulties managing their children’s behavior and 106 from the general population)	Clinical (mothers with problems to cope with their children’s behavior) and community sample	GPS 0.88 to 0.93 PD 0.85 to 0.86 P-CDI 0.86 to 0.91 DC 0.79 to 0.85	NA	NA	NA	BDI-II r = 0.51 B-CPAI r = 0.46 ECBI Intensity r = 0.50 Problem r = 0.54	CFA Three-factor structure supported
SF-3	Wang et al. (2021) [56]	N = 486 (117 fathers and 369 mothers)	Clinical (parents of children with cerebral palsy)	GPS PD 0.83 P-CDI 0.87 DC 0.76	NA	NA	NA	MSPS −0.34	CFA Three-factor structure supported
SF-4	Barroso et al. (2016) [57]	N = 58 mothers and their 12- to 15-month-old infants (predominately Hispanic low-incoming backgrounds)	Clinical (mothers of infants with clinical behavior problems in 3 times assessment)	GPS 0.91 0.92 0.93 PD 0.75 0.71 0.79 P-CDI 0.85 0.87 0.83 DC 0.82 0.81 0.84	0.61 0.66 0.58	NA	0.77 0.78 0.77	CES-D 0.53 PLOC-SF 0.44 ITSEA Externalizing 0.50 Internalizing 0.38 Dysregulation 0.44	NA
SF-4	Touchèque et al. (2016) [41]	N = 318 (4 samples) (Sample 1: 163 mothers, 47 fathers Sample 2: 19 mothers, 19 fathers Sample 3: 18 fathers, 17 mothers Sample 4: 18 mothers, 17 fathers)		GPS 0.89 PD 0.81 P-CDI 0.79 DC 0.79	NA	NA	NA	STAIC −0.37 CDI 0.57 FES 0.70	CFA Three-factor structure supported
LF-4	Çekiç et al. (2015) [58]	N = 386 parents (215 mothers; 171 fathers)	Clinical (parents of children with psychological problems)	DI 0.75 RE 0.56 MO 0.69 AC 0.81 AD 0.74 DE 0.86 TOTAL CD 0.92 CO 0.73 AT0.79 RO 0.79 DP 0.82 SP 0.89 IS 0.73 HE 0.70 Total PD 0.95	0.78	NA	NA	NA	CFA Separately confirmed factor structure for child domain and parent domain.
LF-4	Pereira et al. (2016) [59]	N = 53 mothers of premature infants	Clinical sample	DI 0.88 RE 0.88 MO 0.88 AC 0.89 AD 0.88 DE 0.88 TOTAL CD 0.87 CO 0.89 AT 0.91 RO 0.90 DP 0.88 SP 0.89 IS 0.88 HE 0.89 Total PD 0.88 PSI Global 0.91	NA	NA	NA	NA	EFC Principal components and varimax rotation from 13 subdomains. Two factor structure (child domain and parent domain) are described

(NA) Not Available; SF-3: Short Form 3th version; SF-4: Short Form 4th version; LF-4: Long Form 4th version; PSI-SF (GPS) Global Parenting Stress; (PD) Parenting Distress; (P-CDI) Parent–Child Dysfunctional Interaction; (DC) Difficult Child; PSI-LF Total CD (Total Child Domain); (DI) Distractibility/Hyperactivity; (RE) Reinforces Parent; (AC) Acceptability; (MO) Mood; (AD) Adaptability; (DE) Demandingness; PSI-LF Total PD (Total Patent Domain); (CO) Competence; (AT) Attachment; (RO) Role Restriction; (DP) Depression; (SP) Spouse/Parenting Partner Relationship; (IS) Isolation; (HE) Health. Tools for convergent validation: (GGHQ) Goldberg General Health Questionnaire; (HADS) Hospital Anxiety and Depression Scale; (WHOQOL-BREF) World Health Organization Quality of Life Instrument—Short Form; (ALES) Appraisal of Life Events Scale; (PSDQ) Parenting Styles and Dimensions Questionnaire; (KPSS) Kessler’s Psychological Distress Scale; (CES-D) Center for Epidemiologic Studies Depression Scale; (IOWA) Inattention/Overactivity with Aggression Rating Scale; (PBQ) Parenting Behavior Questionnaire; (SDQ) Strengths and Difficulties Questionnaire; (CBCL) Child Behavior Checklist; (BDI and BDI-II) Beck Depression Inventory; (PSOC) Parental Sense of Competence; (PLOC, PLOC-SF) Parental Locus of Control; (B-CAPI) Brief Child Abuse Potential Inventory; (ECBI) Eyberg Child Behavior Inventory; (MSPS) Multidimensional Scale of Perceived Support; (ITSEA) Infant–Toddler Social and Emotional Assessment; (STAIC) State–Trait Anxiety Inventory for Children; (CDI) Children Depression Inventory; (FES) Family Environment Scale; (ICC) Intraclass Correlation Coefficient; (ISR) Item Separation Reliability; (PSR) Person Separation Reliability; (CFA) Confirmatory Factor Analysis; (EFA) Exploratory Factor Analysis.

## Data Availability

Not applicable.
